# A prospective hazard analysis of real‐time adaptive helical tomotherapy

**DOI:** 10.1002/acm2.70478

**Published:** 2026-01-30

**Authors:** Jonathan Hindmarsh, Scott Crowe, Jemma Walsh, Tanya Kairn, Sonja Dieterich, Jeremy Booth, Paul Keall

**Affiliations:** ^1^ Image X Institute Faculty of Medicine and Health University of Sydney Eveleigh NSW Australia; ^2^ Cancer Care Services Royal Brisbane and Women's Hospital Herston QLD Australia; ^3^ Department of Radiation Oncology UC Davis Health Sacramento California USA; ^4^ Northern Sydney Cancer Centre Royal North Shore Hospital St Leonards NSW Australia; ^5^ Institute of Medical Physics School of Physics University of Sydney Camperdown NSW Australia

**Keywords:** hazard analysis, helical tomotherapy, radiotherapy, real‐time adaptive radiation therapy (ART), safety, system theoretic process analysis (STPA)

## Abstract

**Background:**

Following the release in 2016 of the report of the American Association of Physicists in Medicine Task Group 100, there has been growing interest in the use of prospective hazard analysis in radiation therapy. System Theoretic Process Analysis (STPA) is an emerging technique in this domain that is particularly suited to processes that involve time sensitive collaboration, decision‐making and/or automation.

**Purpose:**

The goal of this research was to use STPA to evaluate existing processes and procedures with an aim to identify improvements, gaps or unforeseen risks stemming from implementing real‐time adaptive treatment on a helical tomotherapy platform.

**Methods:**

The Radixact treatment delivery system (Accuray Inc., Sunnyvale, CA, USA), an evolution of the Tomotherapy platform, incorporates upgrades such as the Synchrony system for real‐time motion monitoring and treatment adaptation. In collaboration with a team from the radiation oncology department of a large public hospital, a prospective hazard analysis focused on the real‐time adaptive capabilities of the Radixact Synchrony system was conducted using STPA. The system boundaries were defined and a control structure model comprising sub‐systems and control actions was developed. Unsafe control actions were identified and broad‐based causal scenarios were generated. The causal scenarios that were novel, specific to Synchrony or challenging to mitigate were selected for further analysis regarding impacts and potential causes, following which mitigation strategies were proposed, taking into consideration the hierarchy of controls.

**Results:**

A control structure model encompassing the entire patient journey was developed, incorporating all the hardware and software components and human decision makers. The model consisted of 12 sub‐systems and 21 control actions, resulting in 108 unsafe control actions and 595 causal scenarios. Sixty‐one causal scenarios were selected for further analysis, for which mitigation strategies were proposed based on the hierarchy of controls. These included the development of better reference documentation, the systematic testing of the sensitivity of tracking performance to changes in tracking parameters, guidance around setting and documenting tracking parameters, and documentation review.

**Conclusions:**

STPA was effectively used to assess the Radixact Synchrony system's real‐time adaptive radiation therapy capabilities, providing insight into how the system could become unsafe throughout the patient journey. While focused on Radixact Synchrony and real‐time adaptive radiation therapy, this study offers a transferable example of STPA application, from analysis initialization to mitigation, that can inform other safety assessments in radiation therapy.

## 1 INTRODUCTION

There has been growing interest in the application of prospective hazard analysis in radiation therapy for the purposes of safety, cost, and resource use optimization. Interest was initially prompted by the formation of American Association of Physicists in Medicine Task Group 100 in 2003 and has steadily increased following the release of the final report in 2016.^1^, ^2^

System Theoretic Process Analysis (STPA) is an emerging prospective hazard analysis tool that has growing applications in radiation therapy, particularly systems that are reliant on time sensitive decision‐making and/or automation. STPA has been used within radiation oncology to assess SRS,^3^ IMRT,^4^ Halcyon,^5^, ^6^ Ethos,^6^ Truebeam,^6^ tomotherapy,^7^ operational decision making,^8^ and patient specific quality assurance.^9^ The study applying STPA to tomotherapy^7^ was performed prior to the release of real‐time adaption on tomotherapy, is focused on preventing the treatment of an incorrect patient and has not made the full analysis available. While these are useful resources, none have considered the impact of real‐time adaption and generally stop short of translating STPA findings into actionable mitigation strategies, leaving a gap in demonstrating the full process from analysis initiation to actional outcomes.

Real‐time adaptive radiation therapy offers the potential for improved targeting accuracy and reduced side‐effects by adapting the treatment to the patient's motion during delivery. This study focusses on the Radixact Synchrony platform, a helical tomotherapy system that enables real‐time adaptive radiation therapy. The system adapts treatment delivery through movable jaws and a binary multi‐leaf collimator (MLC), responding dynamically to detected target motion which is monitored via kV images, supported by surface monitoring for sites subject to respiratory motion.

The Synchrony system on the Radixact, designed for fully automated integration,^10^ was released by Accuray in 2019.^11^ However, while Radixact Synchrony should have minimal impact on processes, the introduction of real‐time adaption into treatment workflows introduces risks that are not well understood or documented. Without structured hazard analysis, these risks may compromise patient safety and treatment efficacy. The tomotherapy platform has been subject to several prospective hazard analyses, they have focused on the pre‐treatment phases,^12^ non‐adaptive treatment delivery,^13^ a rapid palliative workflow,^14^ treating the correct patient,^7^ total marrow irradiation,^15^ and treatment plan execution.^16^ All these studies were either published prior introduction of real‐time adaptive capabilities into the system or did not include such capabilities in their analyses. Given that the addition of real‐time capabilities changes the risks involved in patient treatment, it is important to consider how the processes and procedures around treatment need to be updated to account for these modified risks.

The goal of this research was to use STPA to evaluate existing processes and procedures with the aim of identifying improvements, gaps or unforeseen risks stemming from implementing real‐time adaptive treatment on a helical tomotherapy platform. In doing so, this work contributes to the body of literature on the application of hazard analysis in contemporary radiation therapy and offers a practical example of applying STPA within radiation therapy.

## 2 METHOD

### 2.1 Radixact synchrony

The Radixact treatment delivery system (Accuray Inc., Sunnyvale, CA, USA), an evolution of the Tomotherapy platform, incorporates upgrades such as the Synchrony system for real‐time motion monitoring and treatment adaptation. Real‐time monitoring is achieved by the Radixact Synchrony system through the acquisition of kV images 2–6 times per rotation for all target motion types (typically every 3–6 s), and for targets that experience respiratory motion kV imaging is supplemented with optical surface monitoring.^11^ The system can perform fiducial‐based tracking under both respiratory and non‐respiratory conditions, and fiducial‐free tracking under respiratory conditions.^17^, ^18^ Real‐time adaption is achieved in the cranial‐caudal direction using movable jaws and in the left‐right and anterior‐posterior directions using the binary MLC system. The use of the MLC to adapt the plan makes this the first commercially available MLC tracking system.^19^

### 2.2 System theoretic process analysis

STPA was developed in the late 2000's as a response to the development of complex systems that are not well modeled by the linear chain of event accident models that underpin traditional methods of assessing hazards (such as failure modes and effects analysis (FMEA)).^20^, ^21^ Instead STPA is built on System Theoretic Accident Model and Processes (STAMP)^22^ which uses systems theory to model complex sociotechnical systems. A recent editorial by Wong and Pawlicki notes “the whole FMEA procedure rests on an accident model that is known to have limited effectiveness when applied to complex systems.”^23^ It is for these reasons that STPA was chosen as the prospective hazard analysis method to assess the complex interactions found in real‐time adaptive radiotherapy using the Radixact system.

**FIGURE 1:**
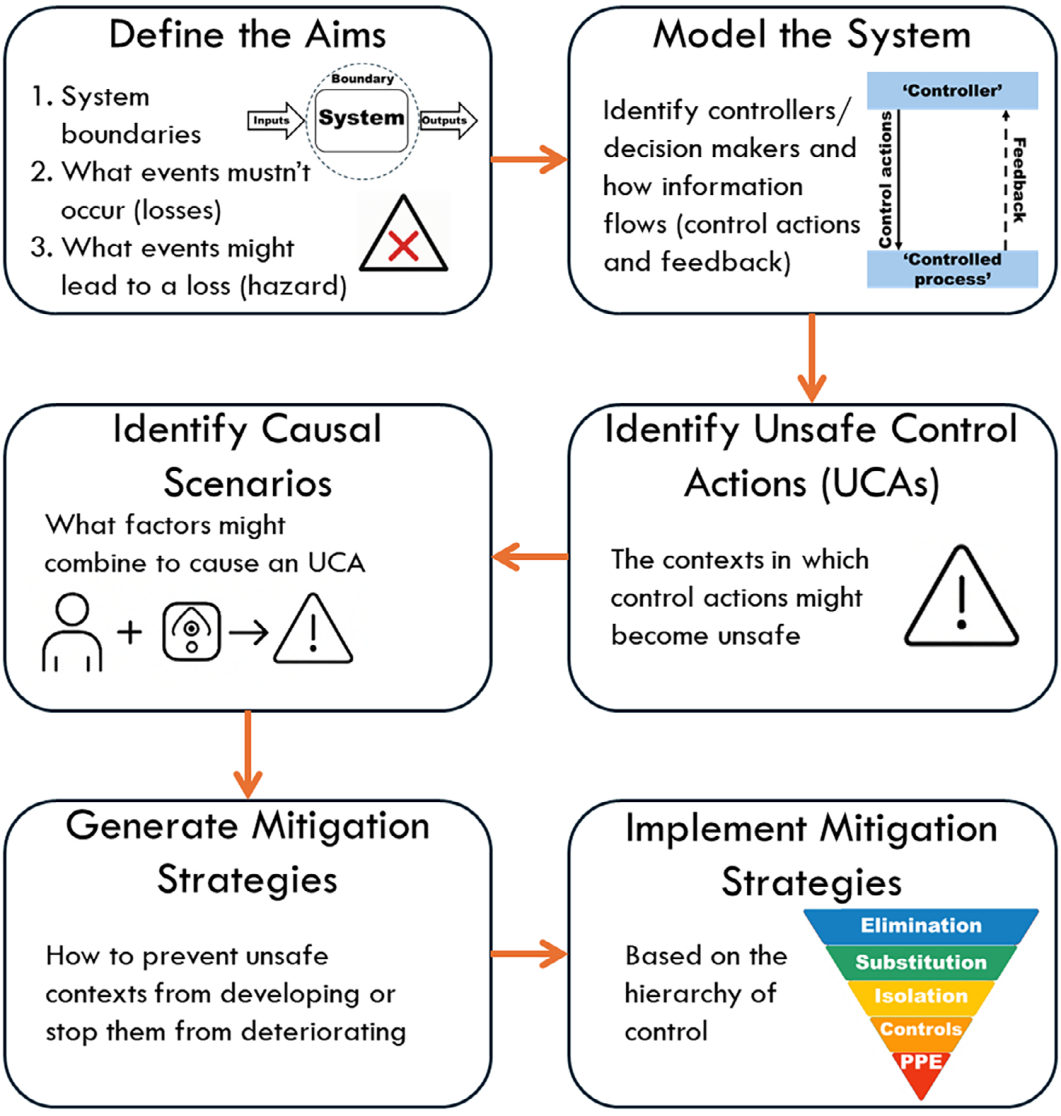
Overview of the steps in performing a System Theoretic Process Analysis (STPA).

The analysis was performed following the STPA handbook^24^ developed by Leveson and Thomas with reference to STPA analyses performed in radiation oncology by other authors.^3^, ^4^, ^5^, ^6^, ^8^ Briefly, the process is described in Figure 1.

STPA models the system using control structures. At a basic level these involve a loop consisting of a controller/decision‐maker, a control action provided by the controller/decision‐maker, a process that receives the control action (typically also a controller/decision‐maker in its own right), and feedback from the process back to the controller/decision‐maker. The method of modeling a system is one of the most significant differences between FMEA and STPA. Where in FMEA, every step of the process needs to be known and understood, in STPA, it is the communication of control and information that is required by the analysis. For more information, readers are directed to a study by Wong and Pawlicki^25^ that goes into detail regarding how to translate a process map to a control structure.

Building a model across all the relevant controllers in the system gives rise to a control structure diagram that details the flow of control actions and feedback within a system. These diagrams are hierarchical in the sense that controllers higher up in the diagram have more generalized responsibilities than those lower down. It is important to note that control actions and feedback pathways can exist independently of the other, and in the initial model used to identify unsafe control actions, the modelling of feedback pathways is not required. Abstraction also plays an important role in the creation of the model, for example, if feedback is ultimately directed to the radiation oncologist, then regardless of how it gets there, it can be modelled as going directly to the radiation oncologist. Similarly, not every sub‐system or control action needs to be included, if, for example, contouring is initiated in an external system and then transferred to the treatment planning system for finalization and approval, these can be modelled as one system or as a feedback pathway from the initiating controller/decision‐maker to the controlled process.

### 2.3 Performing the STPA

The hazard analysis was conducted in collaboration with a team from the radiation oncology department of a large public hospital where two Radixact units had recently been installed. The study focused on evaluating the real‐time adaptive capabilities of the Radixact Synchrony system. Two teams were involved in performing the analysis; the clinical team at the hospital directly involved with the Radixact system and the STPA team who led the STPA. The clinical team consisted primarily of two medical physicists and two radiation therapists and where necessary was expanded to include other professionals working in the department such as radiation oncologists. The STPA team consisted of four medical physicists, all of whom have experience in prospective hazard analysis and an interest in the development and implementation of real‐time adaptive radiation therapy treatments. Table 1 details who was involved in each step. The analysis was documented by the STPA team in the open‐source software Capella^26^, ^27^ using a custom STPA add‐on.^28^ The analysis was distributed using spreadsheets and text documents for collaboration with the clinical team.

**TABLE 1:** Personnel performing each step of the STPA.

STPA step	Directly involved in the creation	Review post creation
1. Define the aims (boundaries, losses, hazards, stakeholders)	STPA Team Clinical team	
2. Model the system (control structure diagram)	STPA Team	Clinical team
3. Identify unsafe control actions	STPA Team	Clinical team
4. Identify causal scenarios	STPA Team	Clinical team
5. Generate mitigation strategies	STPA Team Clinical team	
6. Implement mitigation strategies	Clinical team	

Through an initial series of online discussions between the two teams the analysis was initialized and a model of the system formed (STPA steps 1 and 2). Initialization of the analysis involved introducing the clinical team to STPA, deciding on the losses that must not be allowed to occur, the hazards that could lead to a loss and an initial model of the system. The model of the system was then refined during an in‐person meeting between one member of the STPA team, the whole clinical team and clinical staff.

Taking each of the control actions identified in the control structure diagram, the context under which each action could be unsafe is identified (STPA step 3). The context considers four categories: hazard caused by not providing the control action, hazard caused by providing the control action, hazard caused by providing the control action with wrong timing or out of order, or hazard caused by duration of the provision of the control action (either too short or too long). These are known as unsafe control actions.

Across a further series of on‐line meetings, unsafe control actions were explained and the concept around forming them was discussed between the STPA and clinical teams. During these sessions, initial development of the unsafe control actions was performed. Following the meetings, the STPA team further developed the unsafe control actions and sent them to the clinical team for comment.

Causal scenarios describe the factors that could lead to an unsafe control action and can incorporate the environment, circumstances and conditions in which the control action takes place (STPA step 4). Given there are a multitude of potential causal scenarios for each unsafe control action, for this work, broad scenarios were initially generated by the STPA team for assessment by the clinical team, with the option to add more specificity based on need and interest. Generation of causal scenarios followed the process described by Thomas at the 2024 STAMP workshop^29^ which is an extension of the process described in the handbook. The new method for identifying causal scenarios introduces a framework and formulism that simplifies the process and allows high quality scenarios to be identified.

Prior to formulating the causal scenarios there was discussion between the teams to add more detail to the control structure, particularly regarding the controllers/decision‐maker. For the software and hardware controllers, the role and responsibilities of the component were detailed along with the control algorithm and process model for each of the associated control actions. For the human decision‐makers involved in the system, the role and responsibilities of each were documented along with, for each associated control action, a summary of the process involved in making a decision (including goals, alternatives, methodology, skill classification^30^), what the decision‐maker is expected to know or believe about the system (their mental model) and how they would update their mental model.^31^

This information was used by the STPA team to formulate causal scenarios for all the identified unsafe control actions. Following this the two teams discussed the identified causal scenarios in detail over multiple meetings including how to prioritize them. In STPA, there is not one recommended method of determining which causal scenario to act on, as it is assumed that all causal scenarios could lead to a loss and thus should be mitigated. However, it is accepted that STPA typically generates too many causal scenarios for them all to be mitigated in the first instance, thus there needs to be prioritization of which causal scenarios to focus on.^32^, ^33^, ^34^, ^35^ For this work we chose to prioritize based on how interesting a causal scenario was to the clinical team, where interesting was defined as: not covered by existing processes or checks; novel or specific to the use of the Synchrony system; or challenging to mitigate.

To achieve this interest rating, the STPA team provided the clinical team with a spreadsheet containing all the unsafe control actions along with the corresponding causal scenarios and asked them to indicate (i) whether they considered the scenario relevant, (ii) whether the scenario was interesting on a scale of 1–10 (1 being not interesting, 10 being highly interesting), (iii) whether the scenario was new, and (iv) any comments they had. These responses were used by the STPA team to rank each causal scenario from one to four or uninteresting (where one was very interesting, four was notable and any causal scenario below notable was labelled as uninteresting). The causal scenarios included in the top two ranks were then given further consideration regarding potential impact of the scenario, more detail regarding the cause and potential mitigation strategies first by the STPA team and then refined by the clinical team based on questions posed by the STPA team (STPA step 5). Following this process, mitigation strategies were then proposed based on the hierarchy of controls.^36^, ^37^

The final step of the analysis was a meeting between the clinical team and the STPA team to discuss the analysis, what was learnt from the process, what were the clinical team's takeaways from the process, and what actions they were planning to take.

## 3 RESULTS

### 3.1 Initiation of the analysis

The first two steps in performing an STPA are to determine the losses (those events or conditions that must not be allowed to occur in the system) and the hazards (the events in the system that could lead to a loss). These are displayed in Tables 2 and 3.

**TABLE 2:** Table of losses.

Loss	Name
(L‐01)	Treatment associated harm to the patient
(L‐02)	Non‐treatment associated harm to the staff, visitors or patients
(L‐03)	Damage to equipment
(L‐04)	Reputational damage to the department

**TABLE 3:** Table of hazards and impacted losses.

Hazard	Name	Impacted Losses
(H‐01)	Incorrect patient is treated	[L‐01, L‐04]
(H‐02)	Patient is exposed to the incorrect dose of radiation	[L‐01, L‐04]
↪ (H‐03)	Dose to target	[L‐01, L‐04]
↪ (H‐04)	Dose to designated organ at risk or normal tissue	[L‐01, L‐04]
(H‐05)	Patient treatment is delayed	[L‐04]
↪ (H‐06)	Start date of treatment is delayed	[L‐02, L‐04]
↪ (H‐07)	Delays during treatment	[L‐01, L‐04]
(H‐08)	Mental distress to patients or staff	[L‐02, L‐04]
(H‐09)	Physical Injury to patients of staff	[L‐02, L‐04]
(H‐10)	Radiological injury to staff	[L‐02, L‐04]
(H‐11)	Unnecessary stress on equipment	[L‐03, L‐04]
(H‐12)	Adaption does not improve treatment outcomes or patient quality of life	[L‐04]

### 3.2 Model of the system

**FIGURE 2:**
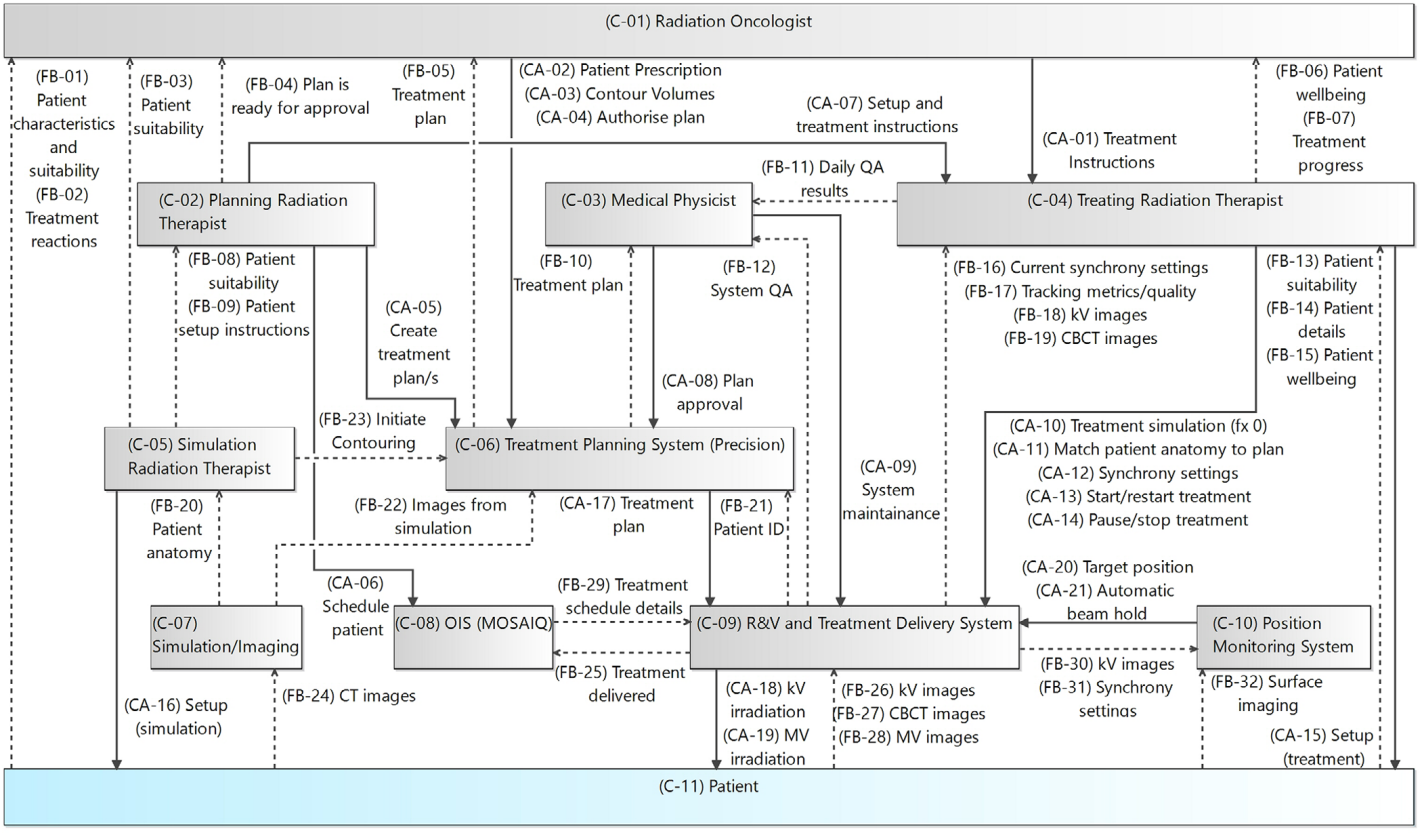
Control structure diagram of the Radixact Synchrony; boxes indicate decision makers/controllers, solid lines indicate control actions (CA), and dashed lines indicate feedback (FB).

To support a comprehensive assessment, the decision was made to model the entire system from radiation oncologist to patient, encompassing all components of both pre‐treatment and treatment workflows (Figure 2). It was also decided to focus on what the local department had control over, and consider the sub‐systems controlled by the vendor as black‐boxes. Where this is most evident is in C‐09 – R&V and treatment delivery system and C‐10 – position monitoring system. Both these controllers are standing in for significant processes, and highly impactful algorithms, however in both cases, apart from settings, they are under the sole control of the vendor and thus the internal workings are not of interest in this analysis.

Although the primary focus of the analysis was the real‐time adaptive functionality of the Radixact Synchrony system, upstream decision‐making during consultation, contouring and planning were recognized as having influence on the quality and safety of adaptive treatment delivery. The control structure diagram was initially developed through two online meetings early in the process followed by iterative refinements via email correspondence culminating in an in‐person meeting several months later. As the teams' understanding of the system evolved, the model continued to be refined throughout the analysis process.

### 3.3 Unsafe control actions

Once the control structure diagram of the system had been developed, unsafe control actions were generated from the identified control actions. From the 21 control actions in the control structure diagram, 108 unsafe control actions were identified. Table 4 is an example of the unsafe control actions identified for the control action “Synchrony settings” (CA‐12) from the treating radiation therapist to the record and verify (R&V) and Treatment Delivery System (the full analysis is included in the supporting material). Generation of the unsafe control actions was done over three online meetings between the STPA and clinical teams and then refined by the STPA team before an in‐person review of the analysis. As with the control structure diagram, as the clinical team grew more familiar with the system, the unsafe control actions were refined to better reflect reality.

**TABLE 4:** Sample table of unsafe control actions (UCA) for the control action CA‐12 (synchrony settings) which goes from the treating radiation therapist to the R&V and Treatment Delivery System.

UCA #	Name	Hazards
*Not providing causes hazard*		
UCA‐58	Treating radiation therapist does not provide Synchrony Settings when plan is loaded for treatment	[H‐05, H‐12]
*Providing causes hazard*		
UCA‐59	Treating radiation therapist provides Synchrony Settings with incorrect model settings/parameters when plan is loaded for treatment	[H‐03, H‐10, H‐12]
UCA‐60	Treating radiation therapist provides Synchrony Settings with inappropriate model settings/parameters during treatment delivery	[H‐03, H‐12]
*Wrong timing or order causes hazard*		
UCA‐61	Treating radiation therapist provides modified Synchrony Settings too late during treatment following frequent beam hold activations	[H‐07, H‐08]
UCA‐62	Treating radiation therapist provides modified Synchrony Settings too early during treatment following beam hold activations	[H‐12]

### 3.4 Causal scenarios

Five hundred and ninety‐five causal scenarios were identified, 176 (29%) were linked to control actions directly connected with the Synchrony system; Synchrony settings (37), kV irradiation (32), MV irradiation (36), target position (28), and automatic beam hold (43). Table 5 provides an example of the causal scenarios identified for the unsafe control action UCA‐58: “treating radiation therapist does not provide Synchrony settings when plan is loaded for treatment”, one of the unsafe control actions identified for the control action CA‐12 in Table 4.

**TABLE 5:** Sample of causal scenarios (CS) related to the unsafe control action UCA‐58: Treating radiation therapist does not provide Synchrony settings when plan is loaded for treatment.

CS#	Name
(CS‐567)	Treating radiation therapist believes that the default settings are appropriate
(CS‐568)	Treating radiation therapist unaware of the need to personalize settings for the patient
(CS‐569)	Plan is not marked as being adaptive
(CS‐570)	Personalized settings are not documented
(CS‐571)	Personalized settings are documented but in the wrong location
(CS‐572)	Synchrony settings revert to default prior to treatment
(CS‐573)	Synchrony settings are updated but not applied/saved
(CS‐574)	Entered settings do not impact underlying processes or implementation
(CS‐575)	Unaware of the importance of using patient specific settings

Following the STPA team formulating the 595 causal scenarios, they presented them to the clinical team for discussion. Following discussion, the clinical team assessed all 595 causal scenarios based on how interesting they were to the clinical team (Table 6). Considering the 61 causal scenarios (10%) that the clinical team found to be interesting and worth investigating further, 27 (5%) were linked to a control action directly connected to the Synchrony system while 34 (6%) were linked to other control actions.

**TABLE 6:** Number of causal scenarios sorted by the STPA team according to how interesting they were to the clinical team.

How interesting?	Number of causal scenarios
1 (very interesting)	17
2	44
3	41
4 (notable)	53
Uninteresting	440

### 3.5 Mitigation strategies

For the 61 causal scenarios given one of the top two rankings (Table 6), further details were added to each causal scenario in the form of potential impact, and why or how the causal scenario might occur considering hardware, software and human factors. Further details were initially added by the STPA team and then refined by the clinical team based on questions posed by the STPA team. An example of the mitigation strategies suggested for very interesting causal scenarios is included in Table 7, for a subset of the causal scenarios identified for the unsafe control action UCA‐58 which are detailed in Table 5.

**TABLE 7:** Sample table of mitigation strategies developed for the interesting causal scenarios (CS) resulting from the unsafe control action UCA‐58: Treating radiation therapist does not provide Synchrony Settings when plan is loaded for treatment.

CS#:	Potential impact	Why/What did not work	Countermeasures/Mitigation strategies
CS‐567: Treating radiation therapist believes that the default settings are appropriate CS‐568: Treating radiation therapist unaware of the need to personalize settings for the patient	Non‐optimal tracking settings Too frequent beam hold Too frequent model rebuilds Too loose beam hold threshold	Default tracking settings inappropriate to patient Treating radiation therapist is unaware of importance of personalizing settings per patient	Investigate impact of settings on tracking quality and create recommendations Training and education for treating radiation therapists regarding tracking settings Develop a quick reference guide for tracking settings so that treating radiation therapists can make quick high‐quality decisions around tracking settings
CS‐570: Personalized settings are not documented	Default tracking parameters used leading to frequent beam holds, frequent rebuilding of motion model, or poor tracking Inappropriate tracking parameters used leading to too loose thresholds, or poor tracking	Patient motion changes between Sim, Fx0 and/or Fx1 Tracking parameters optimized but not documented in an official way Tracking parameters modified but no justification documented	Staff need to be able to identify whether motion has changed since Sim or between treatment sessions Create quick and easy method of documenting parameters including justification that is quickly accessible
CS‐571: Personalized settings are documented but in the wrong location	Default settings used instead of optimized Remembered not optimized settings used	Recording of optimized settings done incorrectly Record of optimized settings misplaced	Create default location for recording optimized tracking parameters (ideally digital and connected with patient record)

### 3.6 Follow‐on from the analysis

The analysis led to the following identified actions:

- Develop better reference documentation resulting from physics commissioning results and initial clinical experience. The purpose would be to communicate the capabilities and limitations of the system to current and future users.
- Systematically test the sensitivity of tracking performance to changes in tracking parameters.
- Create guidance around tracking parameter values for treatment staff, particularly around inappropriate, appropriate and optimal values, when to change and what to change.
- Determine how best to record/document changes in tracking parameters made at treatment.
- Exploration of what can be programmatically extracted from treatment log files to facilitate correlation with tracking parameters.
- Review of the documentation available at the treatment console with the aim of creating usable and accessible documentation treatment staff can easily use.
- Explore the use of treatment console screen recordings to educate staff on motion monitoring requirements during treatment.
- Investigate whether the vendor would be willing to provide additional treatment related information, such as sinograms of the Synchrony treatments, to facilitate assessments of tracking quality.

## 4 DISCUSSION

A prospective hazard analysis of the Radixact Synchrony helical tomotherapy platform has been performed which identified several mitigation strategies, that when implemented would improve the existing processes in the treatment workflow. There were 595 causal scenarios identified across the 21 control actions modelled. The priority of the analysis was to identify gaps that were allowing potentially unsafe conditions to develop; thus, the output of the analysis was focused on those causal scenarios not subject to mature (or any) controls. Of particular interest to the clinical team were the synchrony settings and the impact they can have on the quality of adaption which aligns with a residual risk identified by Accuray in the treatment delivery system manual.

To date there have been seven publications applying STPA to radiation therapy.^3^, ^4^, ^5^, ^6^, ^7^, ^8^, ^9^ The studies by Pawlicki and Wong on the Halcyon,^5^, ^6^ Ethos,^6^ and Truebeam^6^ systems are most similar to this study and their approach informed much of what was done, although their studies stop short of generating mitigation strategies. Due to differences in choices around modelling the control structure diagram which flow onto the rest of the analysis, it is not appropriate to directly compare the studies.

Based on this work, it is possible for a STPA of a radiation therapy system to be performed remotely by a team with experience performing STPA and general experience in radiation oncology in collaboration with an on‐site team. Table 1 details who was involved in each step. It would also be possible for sites wanting a cost‐effective and efficient way to learn to use STPA, to work with an experienced remote facilitator (be that an individual or a team, for example, another site that has experience performing STPA) to initiate and conduct a local STPA without reducing the quality of the outcomes.

Regarding updating the analysis, there are no published guidelines regarding the frequency of updating. For STPA, because the way a control action is achieved does not impact on the analysis, an annual assessment of the control structure model could be performed to check for changes. If there were none, a review of causal scenarios, the resulting mitigation strategies and confirmation they are still valid would be a suitable update process.

### 4.1 Learnings from the causal scenarios and mitigation strategies

The generation of causal scenarios was performed without consideration to current processes or practices (as recommended by AAPM Task Group 100^1^), so many are mitigated via current practices and do not necessarily need further action, hence the large number of causal scenarios categorized as uninteresting. Despite not being a focus of the analysis, a result of not considering current processes in the formulation of the causal scenarios, there is the opportunity to assess current controls for suitability and effectiveness. Not considering current processes also facilitates the identification of redundant checks and overly complex processes. Identifying and removing these can reduce risk and improve efficiency in a system.

There were multiple areas of focus to prevent unsafe actions resulting from the introduction of real‐time adaptive treatment into the department process. The area of focus most obvious is the ability of the system to adapt to motion; however, other areas of focus include treatment instructions, contours, treatment parameters, tracking parameters, radiation therapist decisions around beam on and beam hold, and the amount and quality of feedback provided to the radiation therapist at the treatment unit.

It would be an even larger undertaking to assess all 595 causal scenarios in detail and to come up with potential impact, more detailed explanation, and mitigation strategies for each of them. Hence there was a need to rank and prioritize the causal scenarios. In this work we chose to rank based on interest to the clinical team. Alternatives to ranking based on interest could include (i) ranking based on severity of the potential hazards and losses, (ii) ranking based on immediacy of the hazard in various operational contexts, or (iii) categorizing each causal scenario and using the Pareto principle to select the most impactful categories.^32^

### 4.2 Learnings for future users of STPA

One learning from the process was the way the STPA could be continuously revised throughout the analysis. While in theory performing an STPA is a linear process (as described above), in practice it was found that the analysis would progress, resulting in questions being raised that would lead to refinements and improvements in either the model or the analysis. However, instead of having to redo all the steps further along the process, only those steps directly connected to the changes needed to be revisited, and often only minimal changes were required. This was particularly apparent when changes were made to the model due to either discussions between the teams or better understanding of the system, as following the changes, only those components of the model directly affected needed revising. A consequence of being easily refinable, is a preliminary assessment could be done quickly at the start of a project, and then as experience is developed, the assessment could be refined and improved gradually without having to redo the entire analysis.

It would be possible to adapt the analysis to consider all the functionalities offered of Radixact. To make the analysis applicable to all treatments (adaptive and non‐adaptive) at another site, the process would be to use this work as a template but progress as if starting afresh. The process would be:

- Confirm agreement with losses and hazards
- Check suitability of the control structure model. That is, does the same abstraction apply to the target system? Redesign the model if necessary.
- Are the control actions cross‐applicable?
- For control actions that are the same, confirm agreement with the unsafe control actions
- For new control actions, generate new unsafe control actions
- Update existing causal scenarios and identify new ones for the new unsafe control actions

In STPA, a result of it being a top‐down assessment, all causal scenarios have the potential to result in an unsafe control action, which could lead to a hazard and possibly result in a loss/accident. This has two consequences, first the analysis can quickly become unwieldy, and secondly, it can be difficult to assess all the resulting causal scenarios.

To handle the unwieldy nature of the analysis, it was found that using a software package to collate the analysis was significantly better than using combinations of spreadsheets and text documents. The analysis was initially started in STAMP Workbench^38^ but was transferred midway through the process to Capella^26^ as the custom STPA add‐on^28^ allowed greater refinement and interconnectivity of the analysis with the added benefit of being open‐source.

To keep the number of causal scenarios manageable, it is recommended to keep the losses headlining the analysis specific and confined to those that are unacceptable rather than just undesirable, in order to keep the analysis restricted to scenarios that there is a real need and desire to prevent. Including undesirable conditions is an option; however, it needs to be understood that doing so will increase the scope and time required to complete the analysis. This is a result of the losses and hazards identified in the initialization of the analysis directly influencing the number of identified unsafe control actions and causal scenarios and, to a lesser extent, how useful they are. For example, in this analysis, “mental distress to patients or staff” (Table 3) was included as a hazard that could cause the loss “non‐treatment related harm” (Table 2), meaning that even minor unsafe control actions and causal scenarios that could lead to mental distress should be considered as being unacceptable.

With STPA being such a new technique for hazard analysis, there are developments being made regularly. Published literature is one place to find these developments. An alternative source of information as to the latest developments and how others have approached STPA is via the MIT Partnership for Systems Approaches to Safety and Security website^39^ which has tutorials, links to free resources and slides from past editions of the annual STAMP workshop.

### 4.3 Limitations

This analysis focuses on one implementation of Radixact Synchrony and was primarily performed by the STPA team which was external to the department. This is both a benefit and a limitation, as it enabled a comprehensive analysis to be performed with minimal burden on the clinical team; however, given the scale of the analysis, there is the possibility that there are scenarios that have been overlooked. The analysis also only focused on interactions within the local department and did not consider interactions with the broader hospital, vendors, or regulatory bodies which could provide insight into broader issues around sustainability and efficacy of the real‐time adaptive radiation therapy program.

A limitation to this study is that the prioritized causal scenarios are subjective and biased to the department in question. This was a result of assessing causal scenarios based on their “interest” to the local department, where the two main triggers for interest were (i) how it related to recent experiences on the system, and (ii) perceived potential gap in current controls/mitigations. Assessing the causal scenarios in this way means extending the learnings from this department to others is not possible.

Considering STPA in general, there are three factors that could be considered limitations: the method is unfamiliar, it is a hazard analysis not a risk assessment and a significant time investment may be required to perform an STPA. Considering the method, where FMEA is relatively simple to understand, STPA requires a different way of thinking and approaching the problem of safety that many are not practiced in, meaning learning the method involves a steep learning curve. Secondly, regardless of the method of prioritization, STPA does not consider the likelihood or severity of a scenario occurring, this is an inherent limitation of the STPA method. Thirdly, the time investment required to perform an STPA can be significant. In the case of this analysis, it was the STPA team's second use of STPA and therefore significant learnings were made along the journey. Coupled with the time to learn both documentation software systems and the limited availability of the clinical team, the timeline of this analysis is not a good indicator of how long it would take an STPA to be performed by an experienced team. An estimate of the human‐hours required to perform a similarly comprehensive analysis with an experienced team would be 200–250 h. The most time‐consuming steps of the process were found to be modelling (step 2) and identifying causal scenarios (step 4). Note, this estimate would be strongly dependent on the size of the analysis, the experience of the team and the availability of quality examples in the literature on which the analysis could be based.

## 5 CONCLUSION

This study applied STPA to evaluate the Radixact Synchrony helical tomotherapy system, offering insights into how real‐time adaptive radiation therapy can introduce safety risks both during treatment delivery and throughout the supporting clinical processes. By presenting a complete analysis, from initialization through to the development of mitigation strategies, this work not only highlights potential vulnerabilities but also demonstrates a structured approach to addressing them. While focused on Radixact Synchrony and real‐time adaptive radiation therapy, the methodology and learnings are broadly applicable, providing a practical reference for researchers and clinicians seeking to implement STPA in other radiation therapy contexts.

## AUTHOR CONTRIBUTIONS

**Jonathan Hindmarsh**: Conceptualization, Project Administration, Methodology, Investigation, Data Curation, Writing – Original Draft Preparation; **Scott Crowe**: Conceptualization, Investigation, Writing – Review & Editing; **Jemma Walsh**: Investigation, Writing – Review & Editing; **Tanya Kairn**: Investigation, Writing – Review & Editing; **Sonja Dieterich**: Supervision, Writing – Review & Editing; **Jeremy Booth**: Supervision, Writing – Review & Editing; **Paul Keall**: Conceptualization, Supervision, Writing – Review & Editing

## CONFLICT OF INTEREST STATEMENT

Jemma Walsh has received support from Accuray to attend user group meetings.

## Supporting information



Supporting Information
